# RETRACTION: CircPTPRM Accelerates Malignancy of Papillary Thyroid Cancer via miR‐885‐5p/DNMT3A Axis

**DOI:** 10.1002/jcla.70149

**Published:** 2025-12-09

## Abstract

**RETRACTION**: ChenD. , JiangX. , LuoH. , HuaQ. , and ZhangF. , “CircPTPRM Accelerates Malignancy of Papillary Thyroid Cancer via miR‐885‐5p/DNMT3A Axis,” Journal of Clinical Laboratory Analysis 36, no. 10 (2022): e24688, 10.1002/jcla.24688.36098040 PMC9551131

The above article, published online on 13 September 2022 in Wiley Online Library (wileyonlinelibrary.com), has been retracted by agreement between the journal Editor‐in‐Chief, Rong Fu; and Wiley Periodicals, LLC. The retraction was agreed upon following the authors’ notification to the journal regarding irregularities in the ethical approval procedures for the study. Additionally, third‐party concerns were raised regarding the reuse of the GAPDH panel in Figure 5B, which appears to have been previously published in a different scientific context. Further issues were noted concerning inaccuracies in some of the reported primer sequences. Accordingly, the article has been retracted. The authors have been informed of the retraction decision but were unavailable for final confirmation.

**RETRACTION**: D. Chen , X. Jiang , H. Luo , Q. Hua , and F. Zhang , “CircPTPRM Accelerates Malignancy of Papillary Thyroid Cancer via miR‐885‐5p/DNMT3A Axis,” Journal of Clinical Laboratory Analysis 36, no. 10 (2022): e24688, 10.1002/jcla.24688.36098040 PMC9551131

The above article, published online on 13 September 2022 in Wiley Online Library (wileyonlinelibrary.com), has been retracted by agreement between the journal Editor‐in‐Chief, Rong Fu; and Wiley Periodicals, LLC. The retraction was agreed upon following the authors’ notification to the journal regarding irregularities in the ethical approval procedures for the study. Additionally, third‐party concerns were raised regarding the reuse of the GAPDH panel in Figure 5B, which appears to have been previously published in a different scientific context. Further issues were noted concerning inaccuracies in some of the reported primer sequences. Accordingly, the article has been retracted. The authors have been informed of the retraction decision but were unavailable for final confirmation.

